# Computational Fluid Dynamics Analysis of Blood Flow Changes during the Growth of Saccular Abdominal Aortic Aneurysm

**DOI:** 10.3400/avd.oa.22-00098

**Published:** 2022-12-25

**Authors:** Masanori Murakami, Fei Jiang, Nobuyasu Kageyama, Xian Chen

**Affiliations:** 1 Department of Cardiovascular Surgery, National Hospital Organization, Kanmon Medical Center, Shimonoseki, Yamaguchi, Japan; 2 Department of Mechanical Engineering, Graduate School of Sciences and Technology for Innovation, Yamaguchi University, Yamaguchi, Yamaguchi, Japan

**Keywords:** computational fluid dynamics, saccular abdominal aortic aneurysm, wall shear stress

## Abstract

Computational fluid dynamics analysis of the growth process of saccular abdominal aortic aneurysm was performed. A 3D model of aortic aneurysm was created based on CT images. Properties in terms of wall shear stress, mean flow velocity, mean pressure, energy loss, and pressure loss coefficient were calculated using thermal fluid analysis software “ANSYS CFX.” As the aneurysm expanded, the mean flow velocity decreased and the wall shear stress, mean pressure, energy loss, and pressure loss coefficient increased. Wall shear stress increased when the aneurysm was small, suggesting that is related to the development and growth of the aneurysm. (This is secondary publication from J Jpn Coll Angiol 2021; 61: 3–10.)

## Introduction

Recently, computational fluid dynamics (CFD) has been used in many studies to analyze factors responsible for the development of various vascular lesions from the hemodynamic point of view. A representative example is the numerical analysis to discuss causes of rupture of cerebral arterial aneurysms.^1,2)^ While CFD has been used for other aneurysmal diseases in some reports, there is a paucity of reports using CFD for abdominal aortic aneurysms.

Surgical treatment is recommended for fusiform aortic aneurysms exceeding a certain size, because the expansion rate and the rupture risk generally increases with the aneurysm diameter. Meanwhile, saccular aneurysms are associated with a higher risk for rupture than fusiform aneurysms because expansion of the former type involves protrusions arising from the arterial wall.^3)^ Therefore, saccular aneurysms are often treated as soon as they are found, and their natural course remains largely unknown. Here, we report the construction of three-dimensional shape models from medical images and the CFD analysis of the growth of saccular abdominal aortic aneurysms.

## Subjects and Methods

Diagnostic computed tomography (CT) images of saccular abdominal aortic aneurysms below the renal arteries were used to construct three-dimensional shape models. A general-purpose thermo-fluid analysis software program was used to calculate wall shear stress (WSS), mean intra-aneurysmal flow velocity, mean pressure, energy loss (EL), and pressure loss coefficient (PLc).

### Pre-analysis procedures (Fig. 1)

#### Model construction methods

CT images acquired for preoperative evaluation of saccular abdominal aortic aneurysms below the renal artery level were used to construct three-dimensional shape models. Cases in which the aorta was calcified, but the number of mural thrombi was small were chosen. The models were constructed as follows:

**Figure 1:**
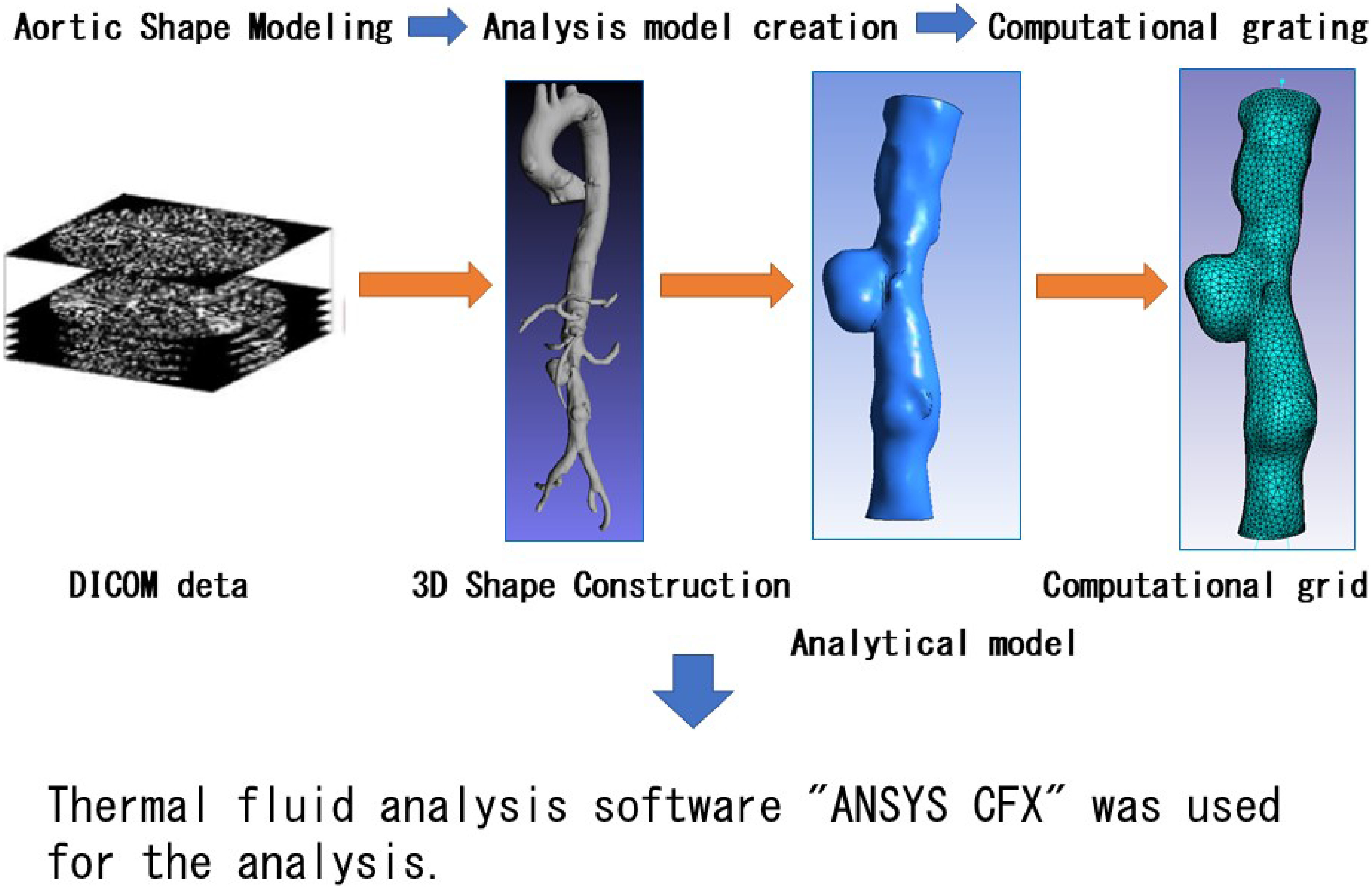
Fig. 1 Steps to analysis.

##### 1. Construction of three-dimensional shape models

Three-dimensional shape models were created from files in the digital imaging and communication in medicine (DICOM) format. The DICOM files were converted to editable high dynamic range image (HDR) files.

The software program used was MRIcro (https://people.cas.sc.edu/rorden/mricro/index.html). Only the aorta was extracted from the HDR files, and three-dimensional shapes were created. The files created were converted to stereolithography files suitable for analysis before saving the data. The software program used was 3D Slicer (https://www.slicer.org/).

##### 2. Construction of analysis models

After noise reduction of the models, the aneurysm sizes were compiled to reproduce the process of aneurysm expansion.

**Figure 2:**
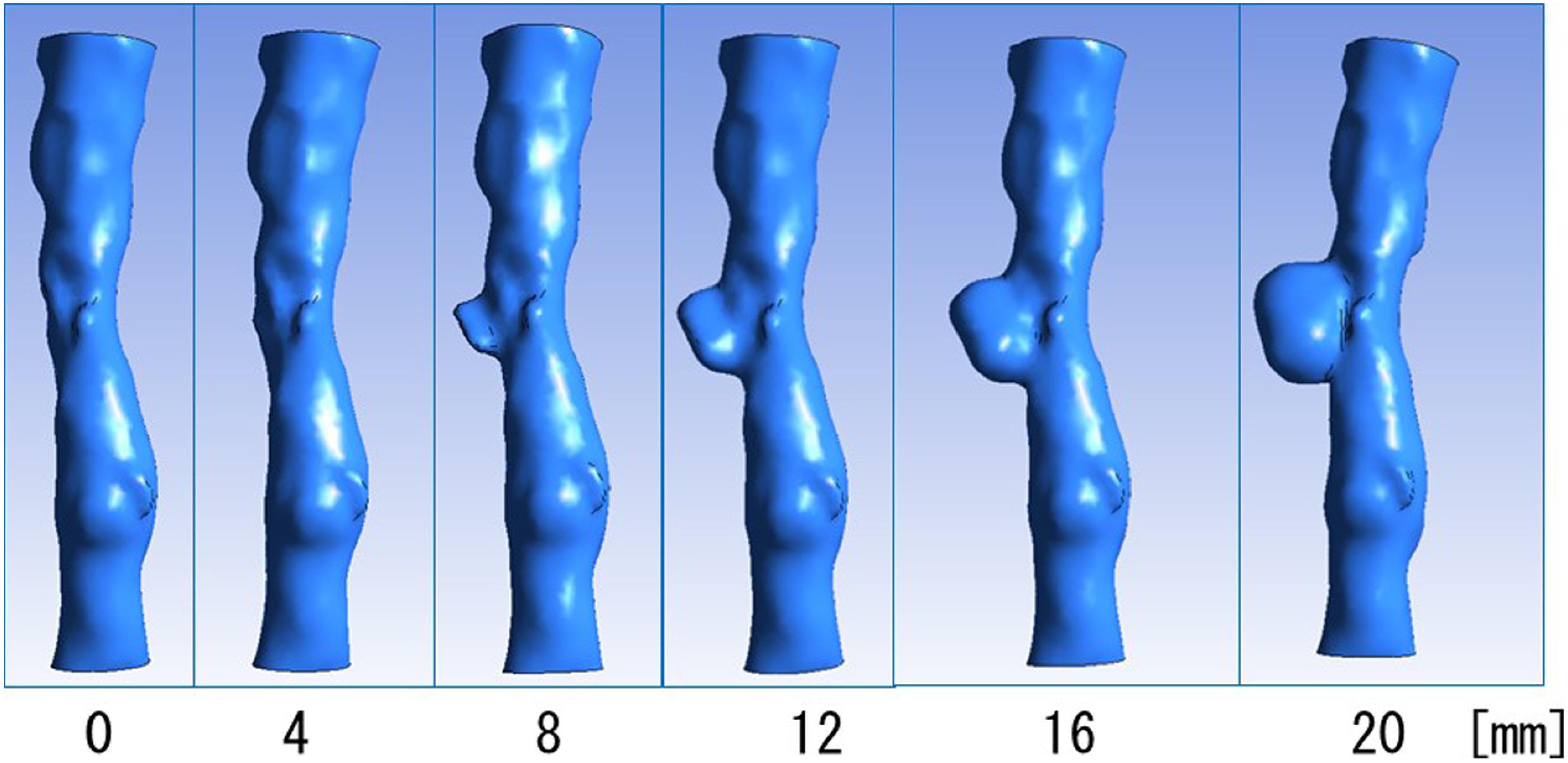
Fig. 2 Size editing of the aneurysm area was performed to reproduce the enlargement course of the saccular aneurysm. Six models with saccular aneurysms of 0 mm, 4 mm, 8 mm, 12 mm, 16 mm, and 20 mm were created.

Since the size of protrusions of saccular aneurysms was approximately 20 mm (the aortic part not included), an aneurysm and the opening were reduced by approximately 20% increments to produce 0-mm, 4-mm, 8-mm, 12-mm, 16-mm, and 20-mm models (**Fig. 2**). For each model, a mesh model was constructed with TetraMesh, which can generate meshes even for complicated shapes. The software program used was Simpleware ScanIP (https://www.jsol-cae.com/product/tool/simpleware/function/).

The resulting 6 blood vessel models (0, 4, 8, 12, 16, and 20 mm) were analyzed under the following conditions.

### Analytical methods

A segment of the abdominal aorta below the renal artery level to the suprabifurcation level was analyzed. Branches were excluded because they have minor effects on the blood flow around the aneurysm. The general-purpose thermo-fluid analysis software program “ANSYS CFX” (ANSYS, Inc., Canonsburg, PA, USA) was used for analysis. Parameters analyzed were WSS, mean flow velocity, mean pressure, EL, and PLc. These parameters were analyzed under the following conditions.

#### Wall shear stress (WSS),^1,2,4,5)^ mean flow velocity, and mean pressure

**Figure 3:**
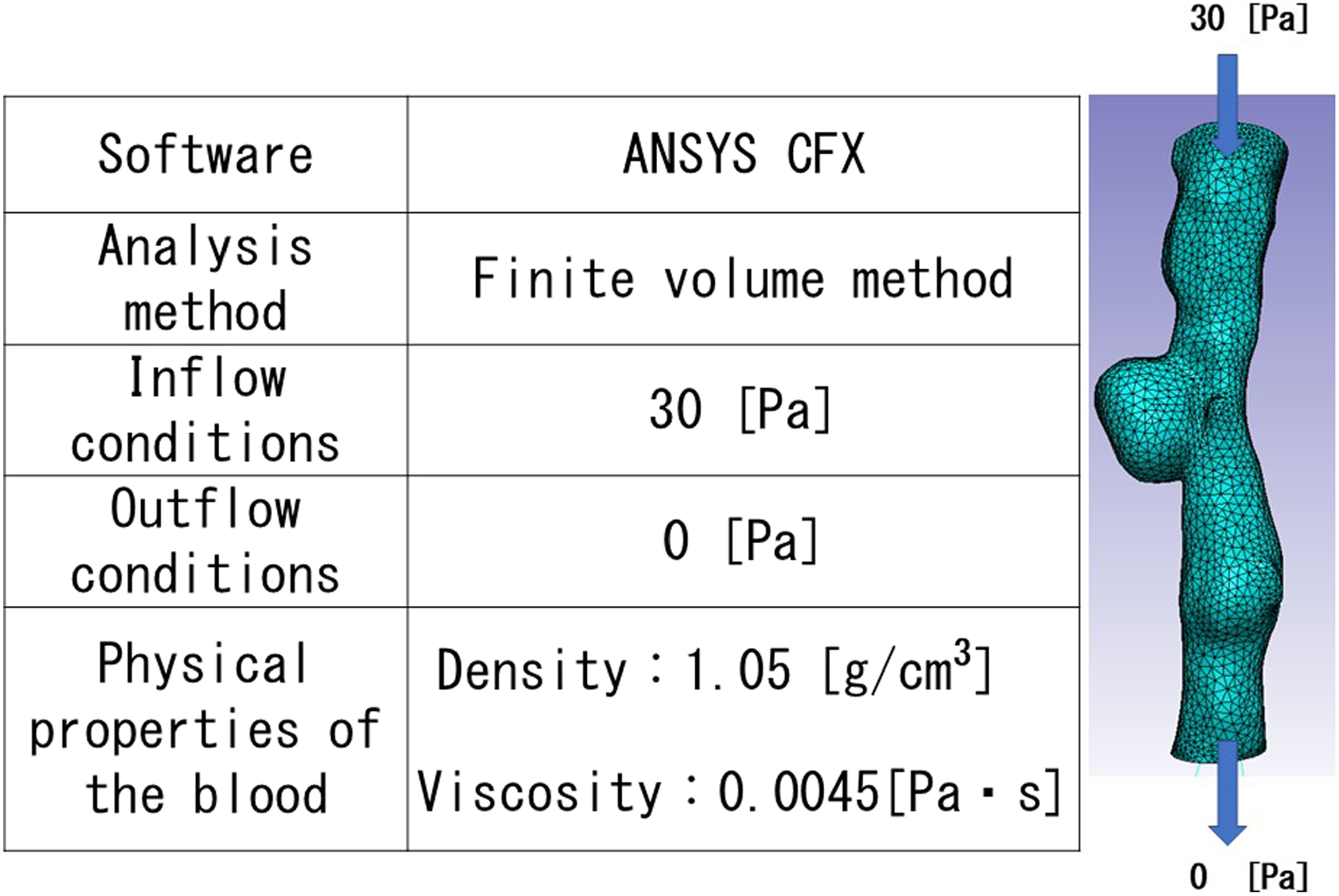
Fig. 3 Analytical conditions for wall shear stress (WSS), mean flow velocity, and mean pressure.

Conditions for calculation of WSS, mean flow velocity, and mean pressure were as follows. The finite volume method (a method of numerical analysis; a region is divided into a finite number of control volumes, and the integral form of a conservative equation for a physical quantity is applied to each volume to calculate the value) was used; and the blood vessel and aneurysm wall surfaces were treated as no-slip rigid walls. The blood flow in the models was a steady flow of an incompressible Newtonian fluid with a density (ρ) of 1.05 [g/m^3^] and a viscosity (*μ*) of 0.0045 [Pa·s]. A pressure difference such that the mean Reynolds number of the model approximated 1000–1250 was applied, because the maximum Reynolds number of abdominal aortic aneurysms is 1250 and the maximum Reynolds number of common femoral arteries is 1000.^6)^ Values obtained were close to assumed values when the pressure difference was 30 [Pa]. Thus, analyses were conducted with pressure values of 30 [Pa] and 0 [Pa] at the inlet and outlet of the model, respectively (**Fig. 3**). The analysis boundary conditions used here assume that the blood pressure is invariable (the pressure at the inlet does not change depending on the aneurysm size). The mean flow velocity and mean pressure refer to space mean values of the models.

#### Energy loss (EL)^7)^ and pressure loss coefficient (PLc)^8)^

**Figure 4:**
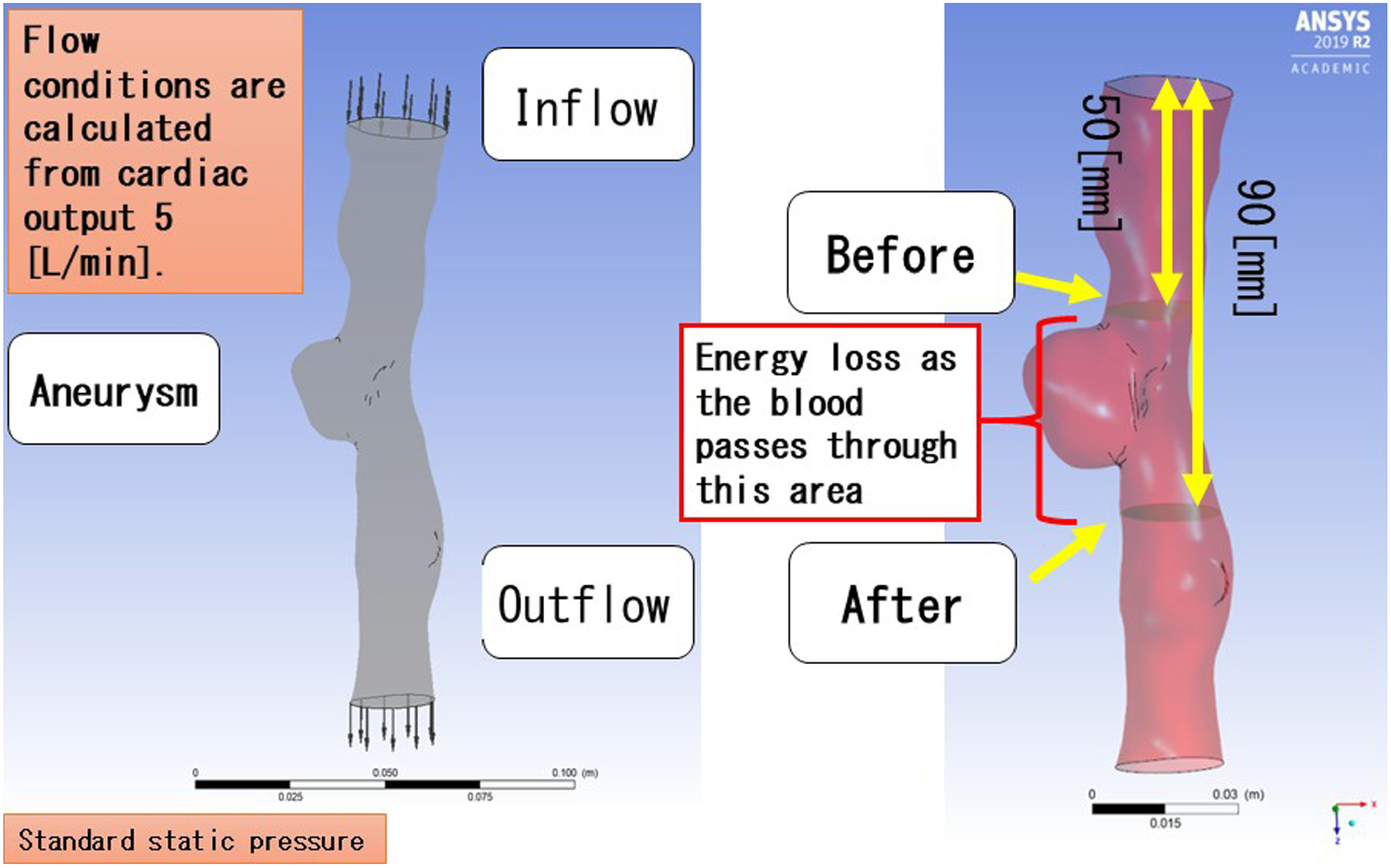
Fig. 4 Analytical conditions for energy loss (EL) and pressure loss coefficient (PLc).

Loss values were calculated for the aneurysm proximal surface located 50 [mm] from the inlet and the aneurysm distal surface located 90 [mm] from the inlet. The blood data used for analysis conditions were same values used for WSS, mean flow velocity, and mean pressure. Assuming a constant flow rate for blood supply from the heart, the inflow and outflow were defined as a flow rate per unit time (5 [L/min] for a common adult cardiac output) and a flow with a static pressure of 0 [Pa], respectively. The EL and PLc between the two surfaces were calculated under these conditions (**Fig. 4**).

## Results

### Analysis results

#### 1. Mean flow velocity distribution

**Figure 5:**
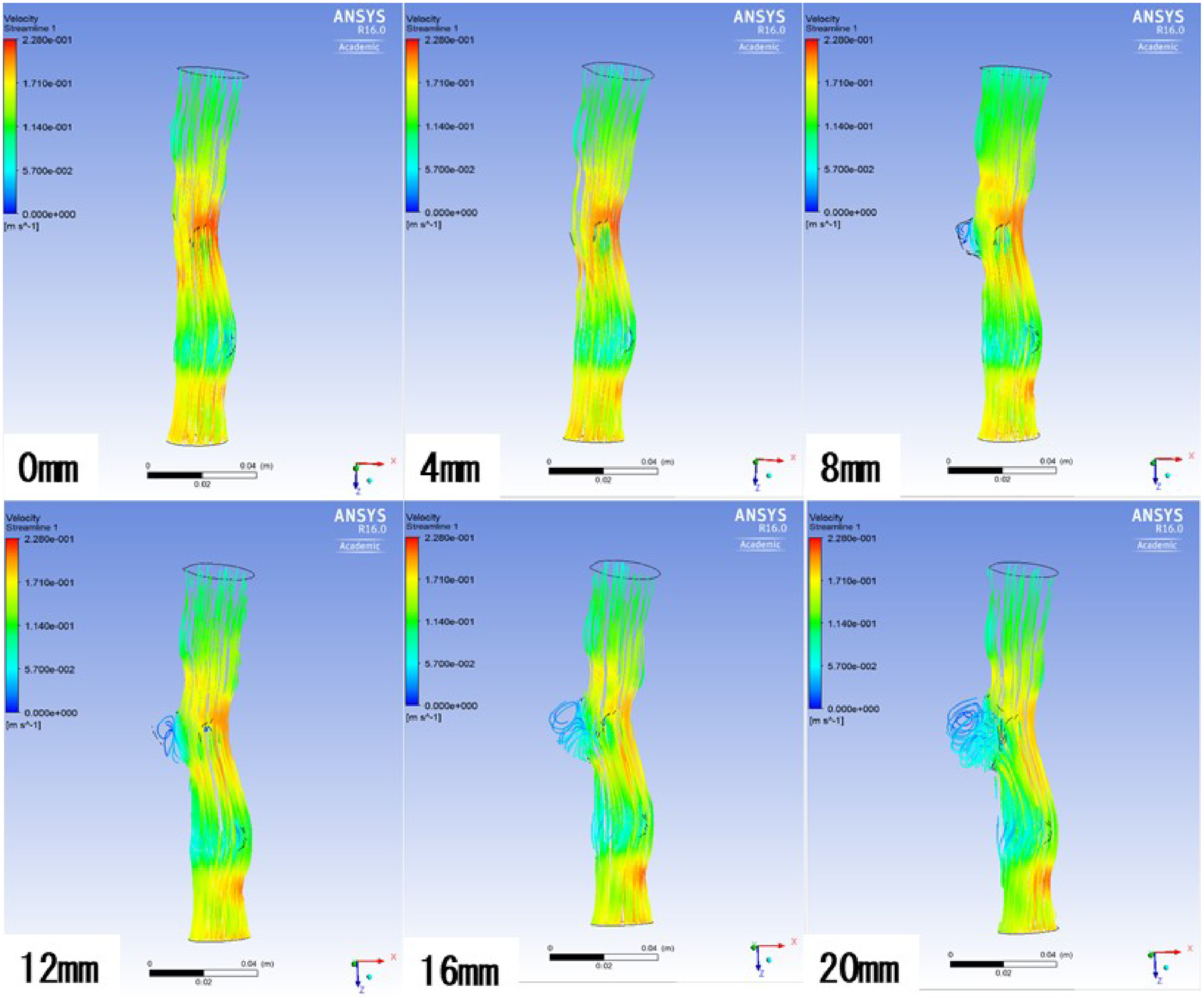
Fig. 5 Flow velocity distribution. As the aneurysm grows, a vortex develops within the aneurysm and the flow velocity around the aneurysm is reduced.

**Figure 6:**
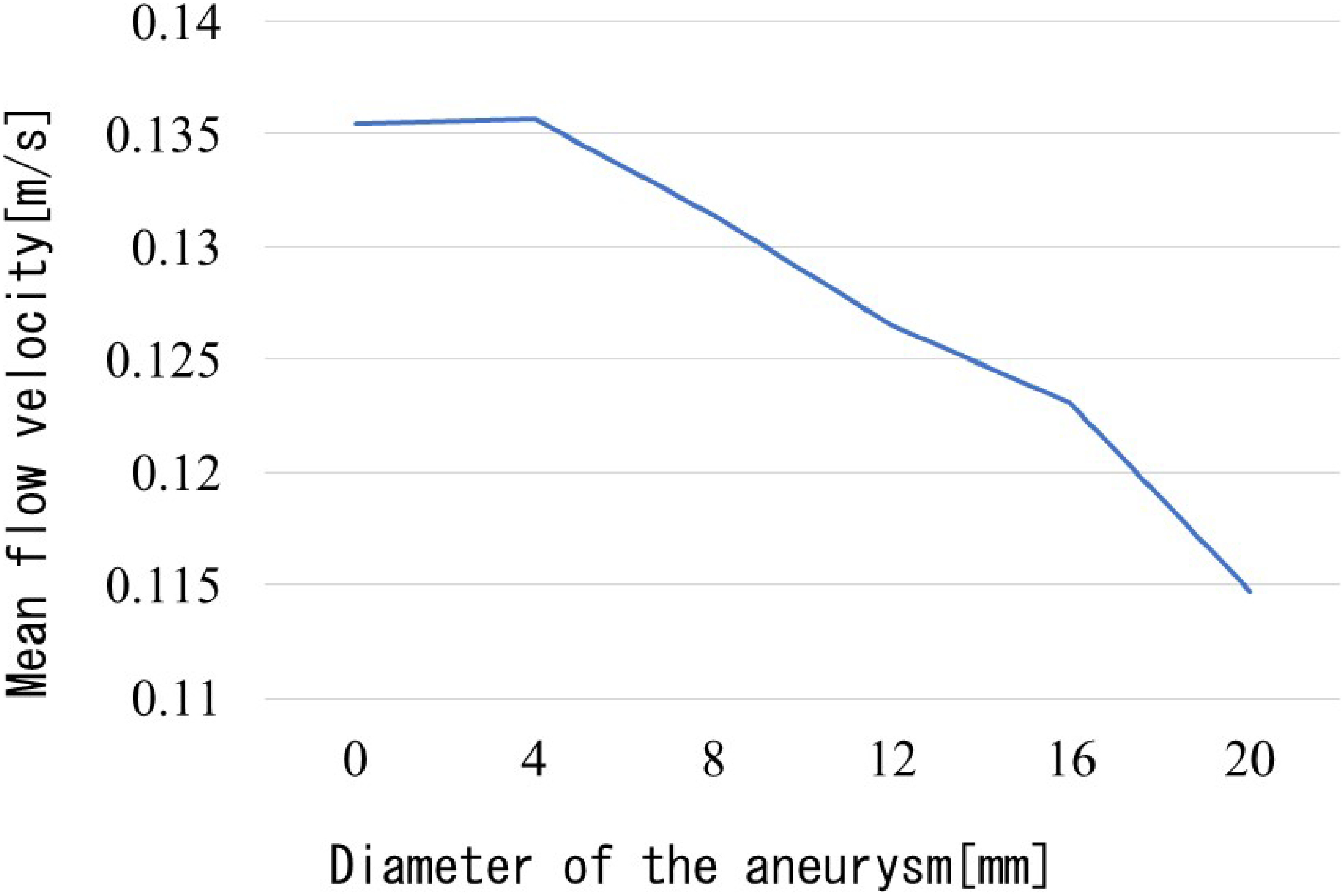
Fig. 6 Mean flow velocity and aneurysm diameter. As the aneurysm expanded, the mean flow velocity decreased slowly.

The mean flow velocity distribution indicates the mean flow velocity in the analytical model. Red and blue denote faster and slower flow velocities, respectively. As the aneurysm size increased, a vortex developed in the aneurysm, and the flow velocity around the aneurysm decreased. The mean flow velocity also decreased gradually as the aneurysm grew. The decrease in the mean flow velocity was particularly large when the aneurysm size increased from 16 mm to 20 mm likely due to a marked increase in the blood inflow into the aneurysm. **Figures 5** and **6** show the mean flow velocity distributions and the relationship between the mean flow velocity and the aneurysm diameter, respectively.

#### 2. Mean pressure and maximum pressure

**Figure 7:**
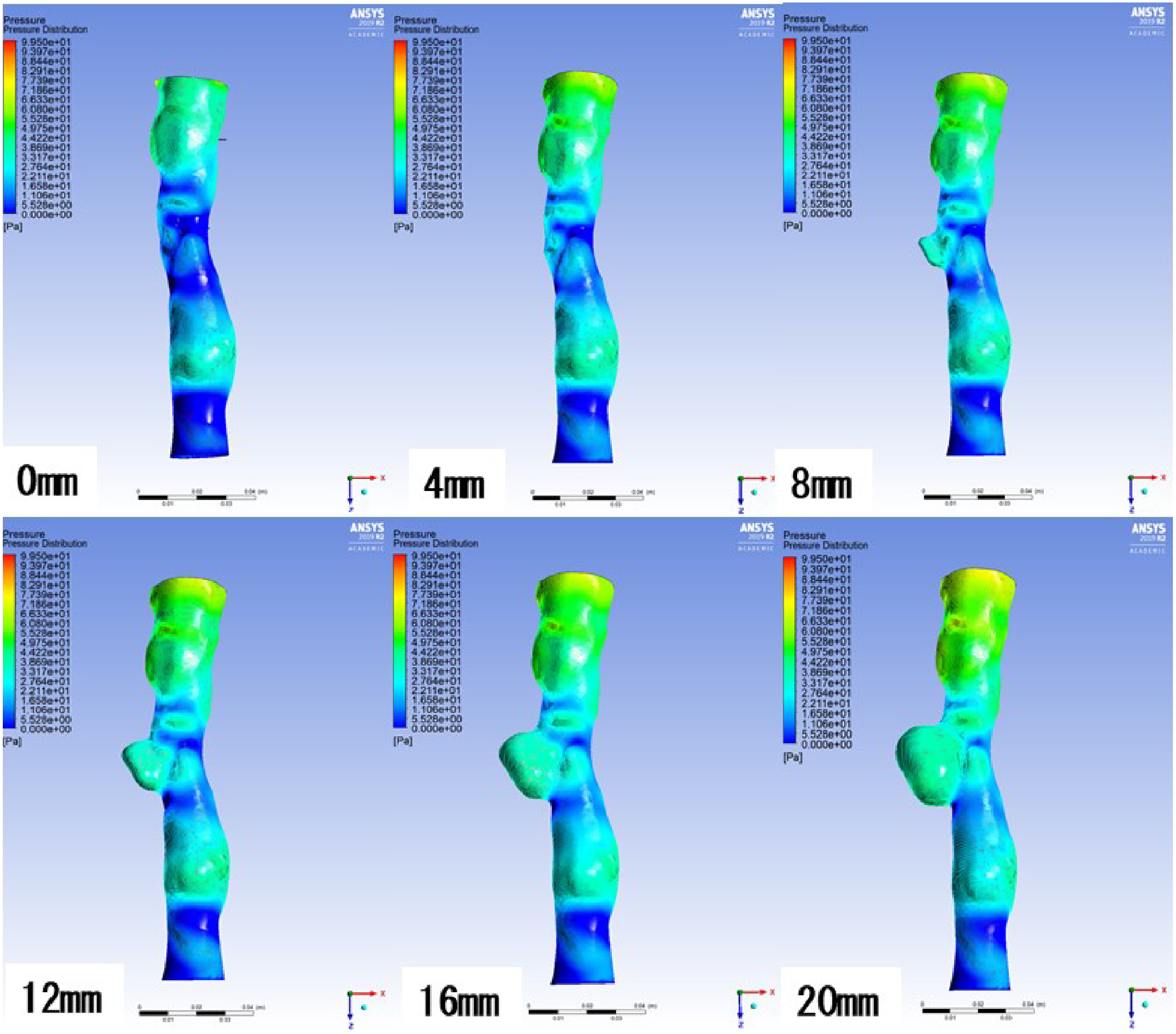
Fig. 7 Pressure distribution. As the aneurysm expanded, the pressure on the wall increased.

**Figure 8:**
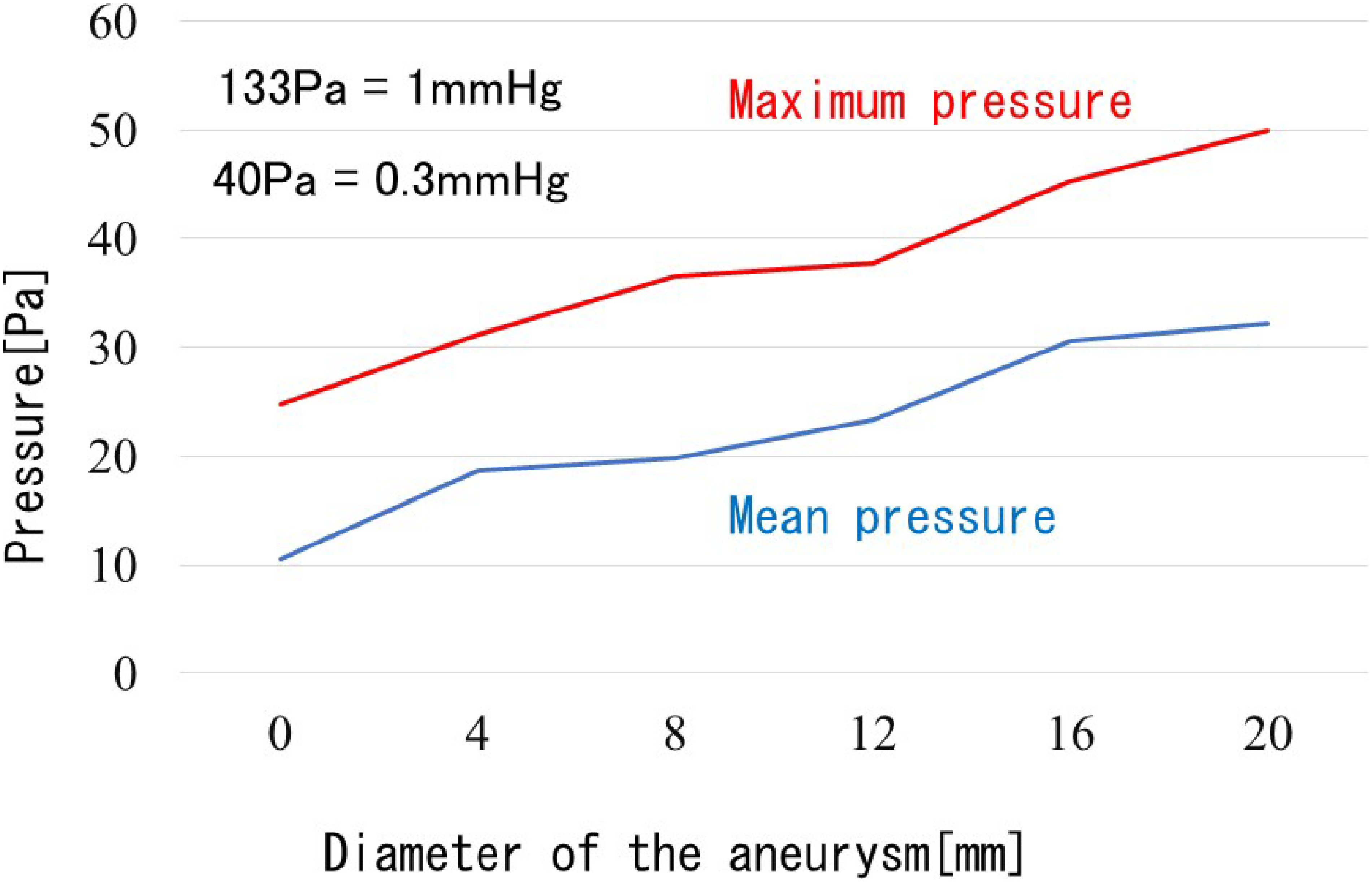
Fig. 8 Relationship between maximum pressure, average pressure and aneurysm diameter. The maximum and mean pressures increased slowly as the aneurysm expanded.

Pressure distribution indicates the pressure on the vessel wall in an analytical model, and the mean pressure and maximum pressure values were calculated. Red and blue denote higher and lower pressure values, respectively. As the aneurysm size increased, the pressure on the wall around the aneurysm increased. Both the maximum pressure and the mean pressure increased gradually with the aneurysm size. **Figure 7** shows the pressure distributions, and **Fig. 8** shows the relationships of the aneurysm diameter with the maximum pressure and the mean pressure.

#### 3. Wall shear stress (WSS)

**Figure 9:**
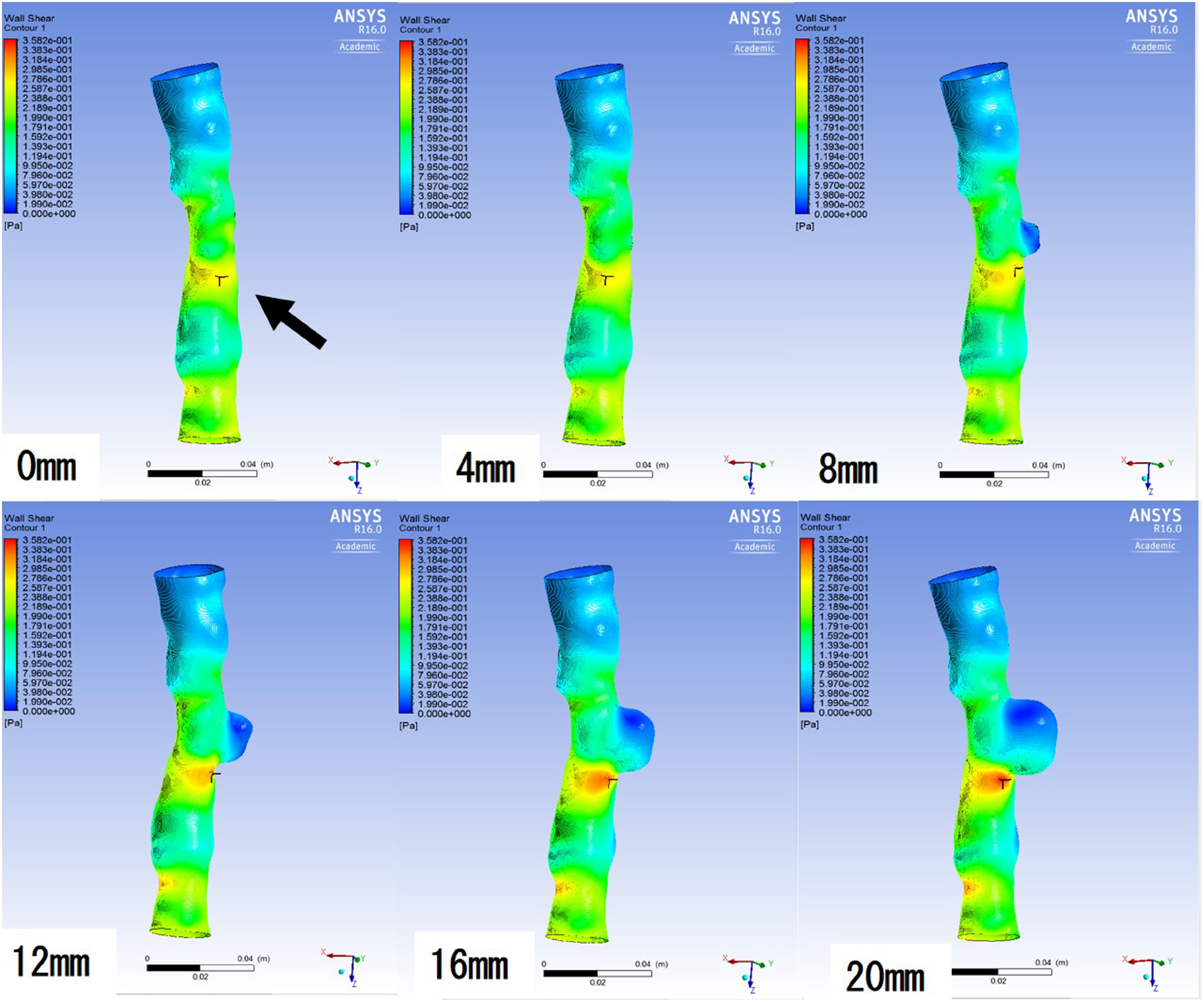
Fig. 9 Wall shear stress (WSS). For all aneurysm diameters, the maximum wall shear stress site was located at the lower edge of the aneurysm (black pointer) and increased as the aneurysm expanded.

**Figure 10:**
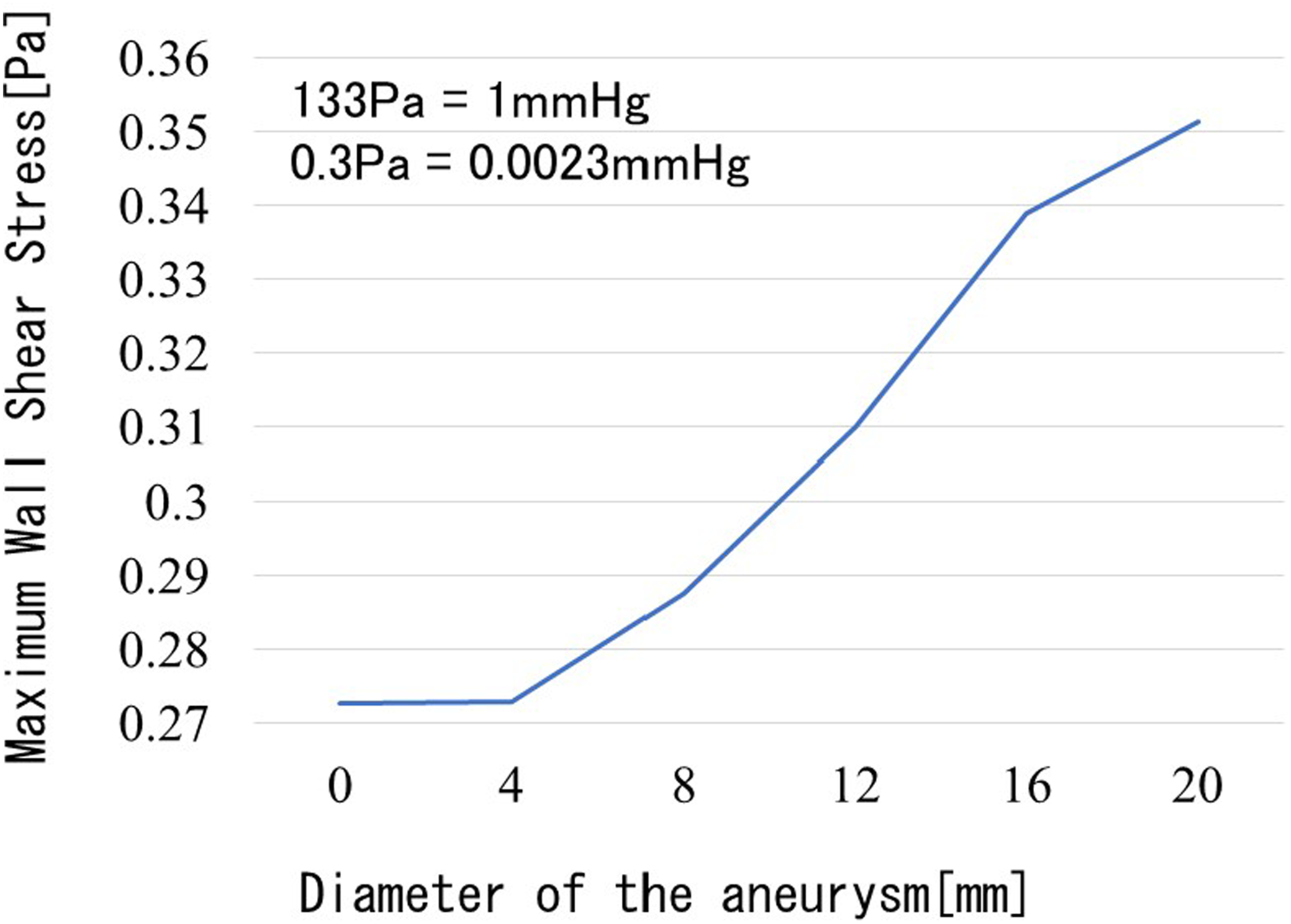
Fig. 10 Maximum wall shear stress and aneurysm diameter. The maximum wall shear stress increased from the time of small aneurysm diameter.

The WSS is a force due to friction between the blood and the vessel wall. In all models, the WSS was maximal at the dorsal lower edge of the aneurysm (a black pointer and an arrow in each panel), and the maximum WSS increased with the aneurysm size. **Figures 9** and **10** show the WSS distribution and the relationship between the maximum WSS and the aneurysm diameter, respectively.

As the aneurysm diameter increased, the pressure on the wall and the maximum WSS increased, while the mean flow velocity decreased. In particular, the WSS started to increase when the aneurysm diameter was small. Moreover, the WSS was low across all areas of the aneurysm wall.

#### 4. Energy loss (EL)

**Figure 11:**
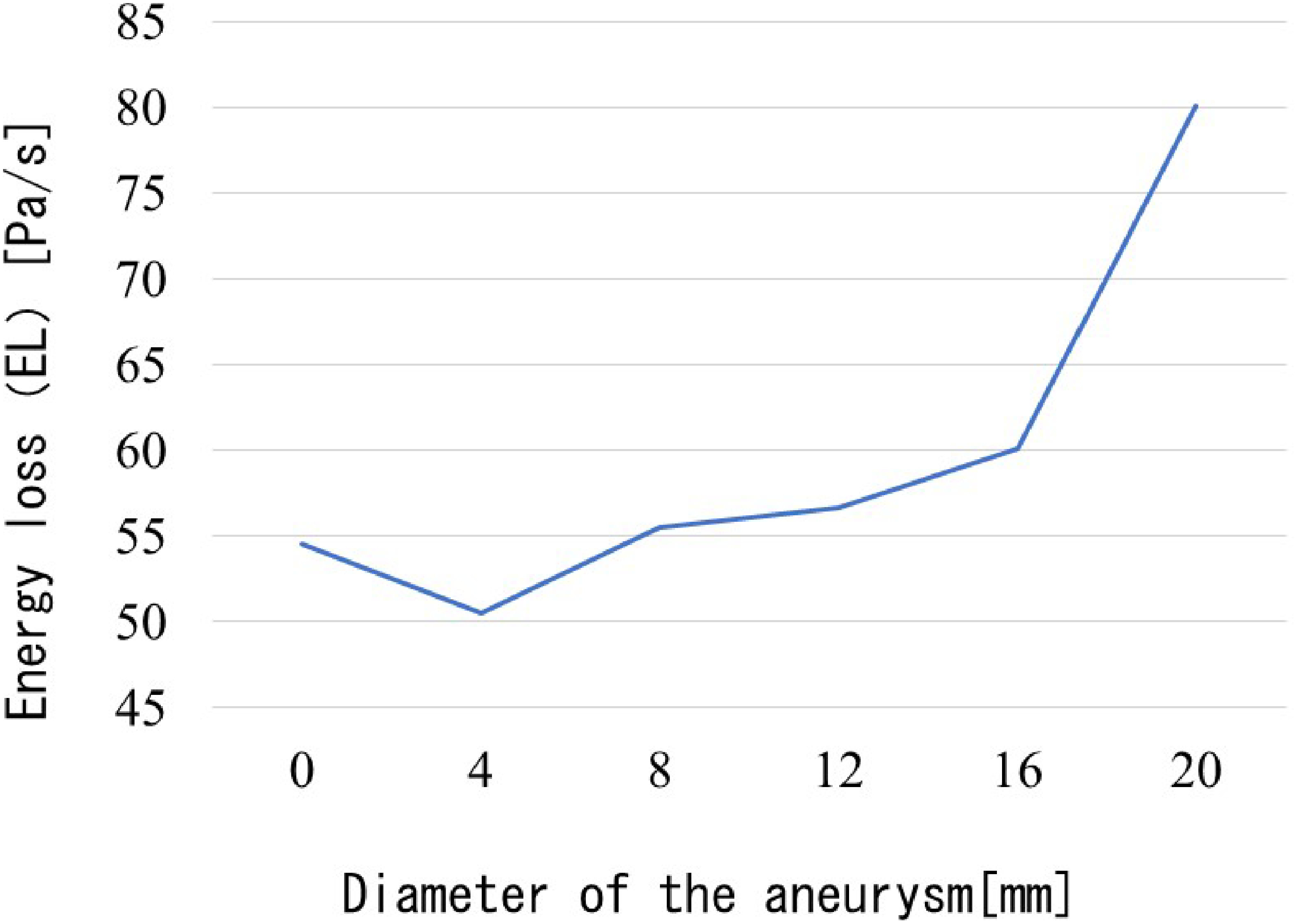
Fig. 11 Energy loss (EL) and aneurysm diameter. As the aneurysm expanded, energy loss (EL) initially increased slowly and then sharply when the aneurysm exceeded 16 mm.

EL is the kinetic energy per unit volume lost due to viscous friction of the blood. As the aneurysm size increased, the EL increased gradually up to 16 mm, beyond which the EL increased sharply. **Figure 11** shows the relationship between the EL and the aneurysm diameter.

#### 5. Pressure loss coefficient (PLc)

The PLc indicates how easily energy is lost depending on the shape of the blood vessel. The PLc is the EL in a specified segment divided by the dynamic pressure (kinetic energy) of the inflow blood vessel, reducing variations due to inflow conditions. A higher PLc means a higher vascular resistance.

**Figure 12:**
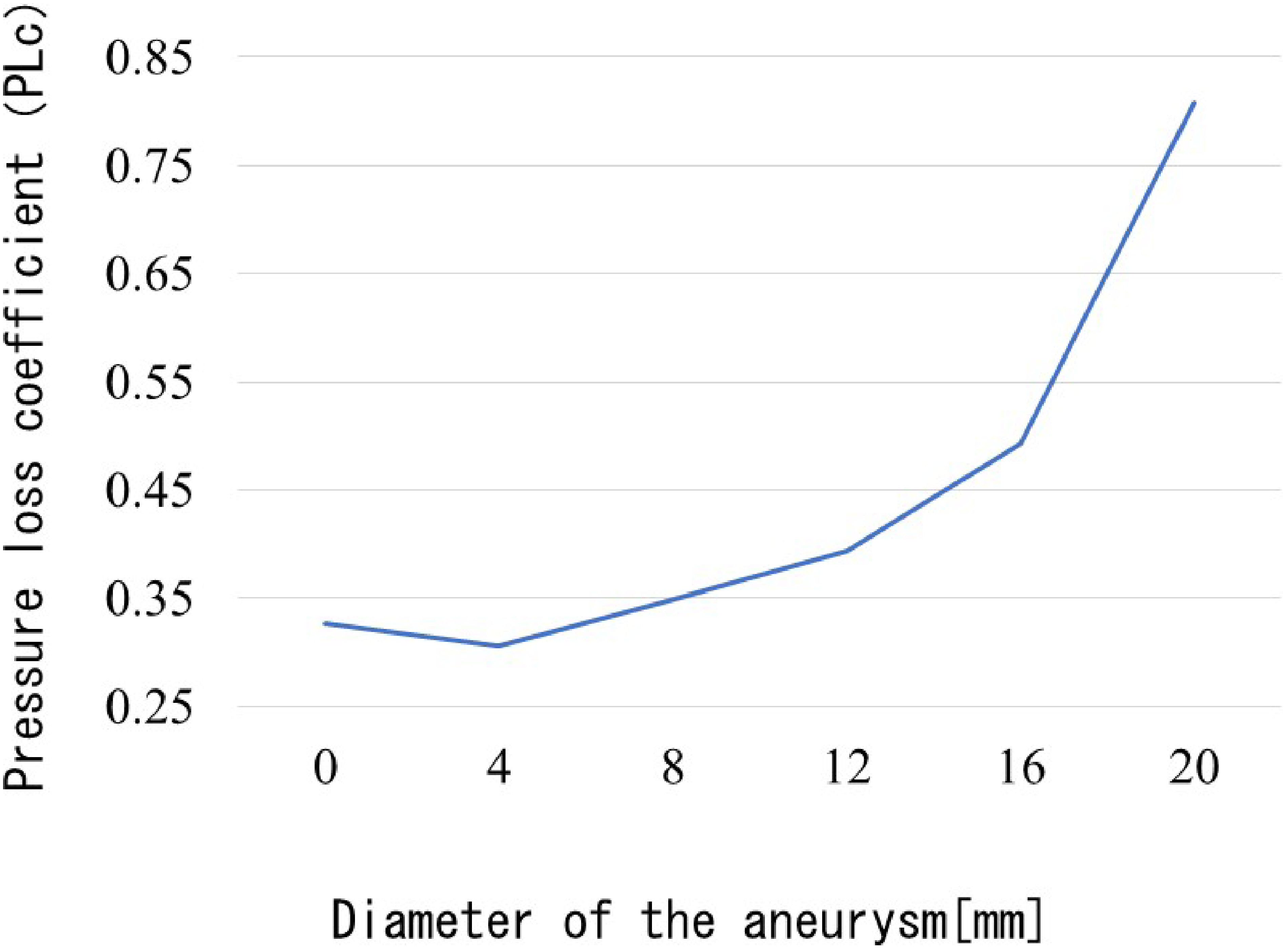
Fig. 12 Pressure loss coefficient (PLc) and aneurysm diameter. As the aneurysm expanded, pressure loss coefficient (PLc) initially increased slowly and then sharply when the aneurysm exceeded 16 mm.

The result was similar to that for the EL. **Figure 12** shows the relationship between the PLc and the aneurysm diameter.

## Discussion

### 1. History of CFD analysis

Steinman et al. first reported the use of CFD in the fluid analysis of cerebral aneurysms using patient-specific shapes in 2003.^9)^ Since then, CFD has been increasingly used in studies to explore for factors responsible for various cerebrovascular lesions from the hemodynamic point of view. While the numerical analysis to discuss causes of rupture of cerebral aneurysms is a representative example in the field,^1,2)^ CFD has also been applied to hydrodynamic studies related to, for example, carotid stenosis, stenting, coil embolization, and sclerotic changes of the cerebral aneurysm wall. Although not as commonly as cerebral aneurysms, aortic aneurysms have also been analyzed with CFD.^10,11)^

### 2. The paucity of reports on analysis of aortic aneurysms (particularly, abdominal aortic aneurysms) with CFD

Virtually no previous studies have described the use of CFD for analysis of abdominal aortic aneurysms. A likely reason is the diversity of components in aneurysms compared to cerebral aneurysms. Abdominal aortic aneurysms are often associated with various calcified lesions in addition to thinning of the arterial wall. Various forms of mural thrombi are also found in aneurysmal lumens, and every case is different from another. While models are constructed based on CT images, the composition and strength of tissue are difficult to estimate accurately based on CT values.

Given the fact that endovascular treatment, in which tissue sample collection is difficult, has become more and more common for abdominal aortic aneurysms, it appears to be almost impossible to develop accurate models. However, we consider that models close to living bodies can be constructed when lesions are minimally calcified and involve a minimal number of mural thrombi, like the models constructed in this study.

### 3. Analysis results

A large number of analytical results on cerebral aneurysms have been reported, showing a certain direction to the mechanism underlying the development, growth, and rupture of cerebral aneurysms. While various parameters have been reported to date,^12)^ no previous articles have described universal parameters that produce similar results. Nevertheless, previous studies have commonly reported that WSS is tightly associated with the growth and rupture of aneurysms.

The parameters measured in this study were: (1) mean flow velocity, (2) mean pressure, (3) WSS, (4) EL, and (5) PLc.

As to the mean flow velocity in the modeled segment, increase in aneurysm size resulted in the development of a vortex in the aneurysm and decreased flow velocity around the aneurysm. The mean flow velocity decreased as the aneurysm size increased. Both the maximum pressure and mean pressure on the vessel wall increased gradually with the aneurysm size. This analysis method assumes an ideal fluid, i.e., steady flow, inviscid, and incompressible. The decrease in mean flow velocity and increase in pressure as the aneurysm size increased can be interpreted with Bernoulli’s principle (for steady flow/inviscid fluid without friction, the sum of energies in the fluid is always constant on the line of flow).

The WSS is calculated from the flow velocity, pressure, and physical properties of the fluid (blood). In this model, the WSS was maximal at the dorsal lower edge of the aneurysm (a black pointer in each panel), and the maximum WSS increased with the aneurysm size. As the aneurysm diameter increased, the pressure on the wall and the maximum WSS increased, while the mean flow velocity decreased. In particular, the WSS started to increase when the aneurysm diameter was small. The WSS is a force due to friction between the viscous blood and the vessel wall, and is proportional to the velocity gradient. While the WSS is a much smaller force than blood pressure (1/100), it is deeply involved in functions of vascular endothelial cells and is considered to be associated with the development, growth, and rupture of cerebral aneurysms.^13–15)^ Even at an aneurysm diameter of 20 mm, the WSS is only 0.351339 Pa=0.002635 mmHg (conversion based on 0.3 Pa=0.0023 mmHg) and is much smaller than blood pressure.

A high WSS has been reported to be involved in the development of cerebral aneurysms, and this link has also been confirmed in animal experiments.^15–18)^ The underlying mechanism is thought to be as follows: a large frictional force is generated where the blood flow forcefully hits the wall, and the resulting WSS promotes the production of metalloproteinases and NO in vascular endothelial cells; these promote vascular wall degeneration and the development of aneurysms.^13,16)^ As a study on cerebral aneurysms, Boussel et al. have reported that a high WSS is involved in the development of cerebral aneurysms, but a low WSS is involved in the growth of cerebral aneurysms.^19)^ While the WSS increases locally in the vicinity of the neck of a cerebral aneurysm, with which the blood flow directly collides, the WSS inside an aneurysm is low, causing proliferation or necrosis of vascular endothelial cells. Consequently, the vessel wall degenerates and grows thinner, and the cerebral aneurysm grows further.^14,20)^

The CFD analysis of saccular abdominal aortic aneurysms conducted in this study also showed that the WSS at the lower edge of the aneurysm neck was high since an early stage of aneurysm formation. Moreover, the WSS on the entire aneurysm wall was low and seemed to be involved in degeneration and thinning of the aneurysm wall. These findings demonstrate the involvement of WSS in the development and growth of arterial aneurysms, as is the case for cerebral aneurysms.

EL is the kinetic energy per unit volume lost due to viscous friction of the blood. We found that the loss of kinetic energy due to viscous friction of the blood was greater than the EL accounted for by the increased area associated with aneurysm growth. The energy lost during passage of blood through an aneurysm is mainly due to friction with the vessel wall and turbulent flow inside the aneurysm. The increased EL associated with larger aneurysm size suggests that friction with the vessel wall and turbulent blood flow inside the aneurysm are increased, and the risk for rupture is elevated. The WSS is an index to evaluate only the friction with the wall. In contrast, EL is a measure reflecting turbulence of blood flow inside the aneurysm as well as friction with the wall. Thus, the EL may serve as a comprehensive index to evaluate the risk of rupture.

The PLc indicates how easily energy is lost depending on the shape of the blood vessel. In other words, a higher PLc means a higher vascular resistance. It is readily conceivable that the PLc, and thus the vascular resistance, increase with aneurysm size.

The following 5 parameters were analyzed: (1) mean flow velocity, (2) mean pressure, (3) WSS, (4) EL, and (5) PLc. Results for (1) mean flow velocity, (2) mean pressure, (4) EL, and (5) PLc were mostly as expected. The maximum WSS started to increase at a smaller aneurysm diameter. Moreover, the WSS at the aneurysm site was reduced, indicating the involvement of WSS in the development and growth of aneurysms. This finding suggests that even small aneurysms have the potential to expand. Aortic aneurysms can also develop just below the renal arteries and at the bifurcation level. Analyses of aneurysms of varying location and shape are expected to shed light on how the WSS, EL, and PLc are involved in the development, growth, and rupture of aneurysms.

### 4. Problems in the present analysis and future prospects

#### a. Model construction

The first problem requiring a solution is the complexity of model construction. A lot of time and effort are required to extract the aorta from CT images in the DICOM format one by one and construct an analysis model. Too much time is required to increase the number of cases and accumulate data with the current method. Artificial intelligence-based methods of model construction have been attempted recently but are not yet practical. There is also room for improvement in accurate replication of the biological environment.

It is possible to construct models accurately replicating aneurysms with minimal calcification and mural thrombi, like the models constructed in this study. It is not possible to construct models accurately replicating all abdominal aortic aneurysms. We expect that the balance between benefits of simplification and benefits of increased complexity to reproduce the biological system more accurately will become clear, and data selection will become easier when the number of cases has increased.

#### b. Analytical methods

For the purpose of accurate blood flow simulation, it is ideal to use patient-specific data for setting of all parameters. However, this is difficult, and mean values in humans are used for actual analyses. CFD analyses with mean data do not reproduce blood flow characteristics in individual cases, which reflect effects of blood pressure and heart rate. Here, the vessel wall and the aneurysm wall were treated as no-slip rigid walls (hard, non-deformable walls), and the blood data were treated as a steady flow. These are also deviations from biological conditions. Obviously, the actual vessel wall is pulsating and varies in thickness depending on the site of measurement. Currently, our group treats the blood flow as a pulsatile flow and the blood vessel as an elastic body in analysis. The establishment of an analytical method incorporating vessel wall motion is awaited to allow for analysis of the growth of aneurysms.

While various parameters have been reported for CFD analysis of cerebral aneurysms, no previous articles have described universal parameters that produce similar results.

It is desirable to standardize parameters and analysis methods.

#### c. Future prospects

CFD techniques are potentially useful for prediction of the course and rupture of abdominal aortic aneurysms. They also hold the potential of contributing to the development of new therapeutic methods. Aneurysm growth may be decelerated by using a device like Flow Diverter, which is used for the treatment of cerebral aneurysms, to prevent the WSS from increasing while the aneurysm is small. Moving forward, we expect that analyses of many cases with different morphologies will reveal the pathophysiology of abdominal aortic aneurysms.

Aside from CFD used in this study, four-dimensional (4D) flow magnetic resonance imaging (MRI) with a MRI instrument is available for blood flow dynamics analysis of large vessels. Both methods can be used to measure the flow velocity, WSS, EL, and other flow parameters in blood vessels. CFD has higher spatial and temporal resolution profiles and produces more reliable results than 4D flow MRI, but is not based on actual blood flow measurements. 4D flow MRI has a lower spatial resolution than CFD, but can use actual measurement data for analysis. There is a high correlation between CFD and 4D flow MRI, and these two methods each have advantages and disadvantages.

These two methods are expected to serve complementary functions in the future.

Furthermore, aside from three-dimensional data-based structural information, various types of information are available, including vital signs (such as heart rate and blood pressure), blood biochemistry test information, blood vessel wall information, environmental factors, and genetic information. These should be combined to make comprehensive decisions. We expect that big data collected, which have been collected through the use of information technology in medical care, and artificial intelligence will contribute to determination of new treatment indications and the improvement/development of treatment methods for abdominal aortic aneurysms.

## Conclusion

As mentioned above, CFD has various issues that require solutions. However, information from computational fluid dynamics can be used as a new piece of medical information by doctors who understand it accurately. The study of abdominal aortic aneurysms has just begun. We believe that new findings may be obtained through continued studies in an increasing number of cases.

## Notes

Conference presentation: The abstract of this article was presented at the 60th Annual Meeting of Japanese College of Angiology (October 2019, Tokyo).

Approval from the Institutional Review Board: This study was conducted with an approval from the Institutional Review Board of our hospital. Approval number (R0202-2).

## Notes

This is secondary publication from J Jpn Coll Angiol 2021; 61: 3–10.
